# Cytological and molecular diagnosis of solid variant of papillary thyroid carcinoma: A case report

**DOI:** 10.1186/1742-6413-5-2

**Published:** 2008-03-19

**Authors:** Giancarlo Troncone, Maria Russo, Umberto Malapelle, Marina Accardo, Angelo Ferraro, Immacolata Cozzolino, Lucio Palombini

**Affiliations:** 1 Dipartimento di Scienze Biomorfologiche e Funzionali, Università degli studi di Napoli Federico II, via Pansini n°5, 80131 Naples, Italy; 2 CEINGE, Biotecnologie Avanzate, via Comunale Margherita 482, 80145 Naples, Italy; 3 Dipartimento di Medicina Pubblica Clinica e Preventiva, Seconda Università degli Studi di Napoli, piazza Miraglia, 80100 Naples, Italy; 4 NOGEC (Naples Oncogenomic Center)-CEINGE, Biotecnologie Avanzate-Napoli, & SEMM – European School of Molecular Medicine – Naples Site, via Comunale Margherita, 482, 80145, Naples, Italy

## Abstract

Papillary thyroid carcinoma (PTC) composed by predominant solid areas is diagnosed as a distinct variant on histological samples. Here we present a case of PTC recognized preoperatively by fine needle cytology as a solid variant. This diagnosis was made by combining cytology with the detection of the BRAF^VK600-1E ^mutation, the molecular hallmark of the solid variant of PTC. Histological and molecular evaluation of the surgical specimen confirmed this pre-operative diagnosis. Thus combining cytology to *BRAF *molecular analysis is useful to refine the cytological diagnosis of this variant also on FNC specimens.

## Introduction

The solid variant of papillary thyroid carcinoma (PTC) is dominated by solid sheets of tumour cells showing typical nuclear features [1]. Since this sub-type of PTC is a rare and poorly characterized, there is disagreement on its behaviour [2]. An aggressive course may be suggested by its association to vascular invasion and extra-thyroidal extension in about one-third of cases [1]. Nikiforov *et al *showed a slightly higher frequency of distant metastases and less favourable prognosis than classical PTC [2]. Thus, the pre-operative sub-typing of PTC may help in refining the appropriate therapeutic strategy and in leading to a more aggressive surgery.

Molecular tests are increasingly being applied to thyroid fine needle cytology (FNC) samples as additional diagnostic tools [3]. In particular, mutations of the *BRAF *kinase gene are highly prevalent and specific for PTC [4]. Thus, their detection is very useful to refine the pre-operative diagnosis of PTC in those cases that are indeterminate on FNCs [5-10]. In addition, analysis of a large series of histological samples of PTC has shown a close relationship between PTC typing and some *BRAF *mutations, which can be exploited to specify the variant of PTC preoperatively [11]. In fact the most frequent *BRAF *mutation, the V600E amino acid change, is associated to a papillary pattern of growth in both conventional and variant (tall cell, Warthin-like and oncocytic) PTCs, whereas the much less frequent K601E mutation has only been observed in the follicular variant of PTC [11]. In addition, a triplet deletion of the *BRAF *gene leading to the replacement of a valine and a lysine by a glutamate (BRAF V600E + K601), has been associated only to the solid variant of PTC on histological samples by *Trovisco et al *[12]. This latter relationship has been confirmed in a recent investigation by *Lupi et *al; this latter study also showed the very low frequency of this event, that occurred in one case out of 500 PTCs histologically examined [13]. The association between the BRAF V600E + K601 and PTC solid variant is further confirmed by this present case; the interest relies on the fact that the correct diagnosis of solid variant of PTC was made preoperatively by combining the cytological evaluation to the molecular detection of the BRAF V600E + K601 mutation on FNC samples.

## Case presentation

A 64 year old man presenting with a 17 mm single solid nodule of the right thyroid lobe underwent fine needle biopsy under ultrasound guidance. Two smears prepared for standard cytological assessment were Diff-Quick^® ^stained and evaluated on-site. The immediate microscopic observation of highly cellular smears prompted the performance of an additional pass; the obtained material was suspended in 300 μl of RNAlater^® ^(QIAGEN Inc.) for molecular analysis of the BRAF mutational status.

**Figure 1:**
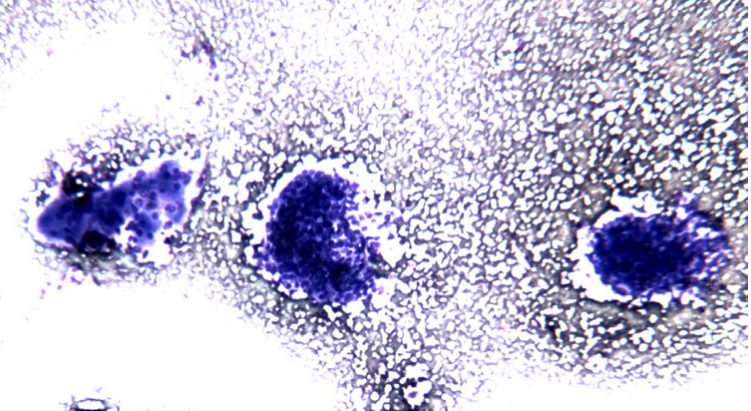
FNC sample of a solid variant of PTC. At low power, in a clean background solid aggregates of epithelial follicular cells arranged in syncytial-type tissue fragments are observed (Diff-Quick^® ^staining 4×)

**Figure 2:**
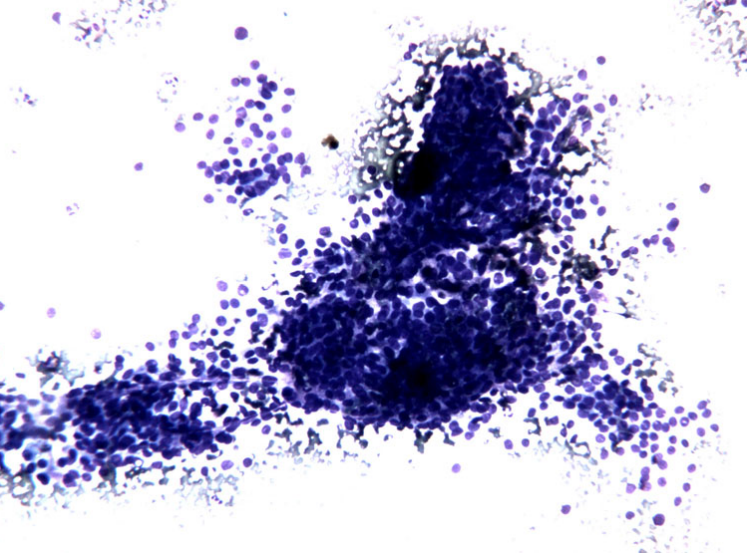
FNC sample of a solid variant of PTC. Microscopic field containing monolayered epithelial cellular sheets. Note the occurrence of nuclar overlapping and crowding and also of a certain degree of variability in cellular shape and size with anisocytosis and anisonucleosis. (Diff-Quick^® ^staining 10×)

**Figure 3:**
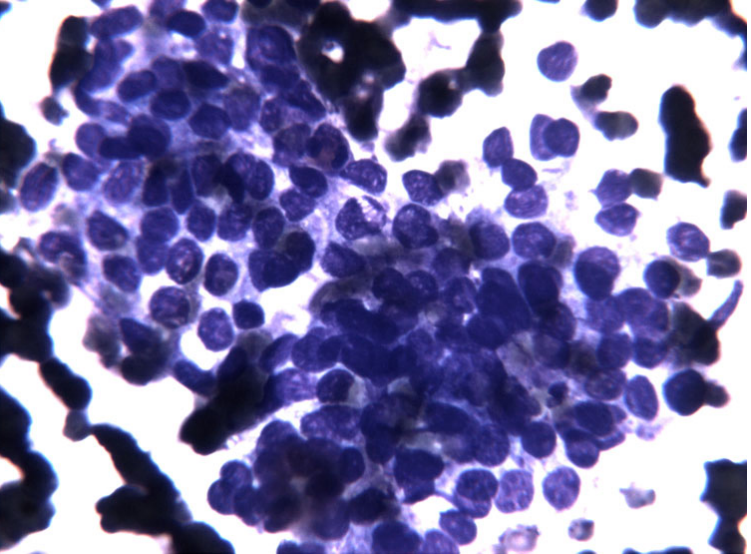
FNC sample of a solid variant of PTC. At higher magnification note the presence of enlarged nuclei with fine chromatin, with occasional nuclear grooves and micronucleoli. These changes prompted the performance of a second pass to perform *BRAF *molecular analysis. (Diff-Quick^® ^staining 40×)

The smears showed, in a clean background with only a little amount of thick colloid, an elevated cellularity composed by neoplastic epithelial follicular cells prevalently arranged in syncytial-type tissue fragments (Figure 1); the cells present at the periphery of these groups often showed overlapping and crowding (Figure 2). Less frequently the epithelial follicular cells were organized in monolayered sheets; the cells in these groups showed a certain degree of variability in cellular shape and size with anisocytosis and anisonucleosis (Figure 3). The nuclear changes included grooves and micronucleoli, whereas intranuclear inclusions were not observed. Since these cytological features were not fully diagnostic but only suspicious for PTC, the molecular analysis of the aspirated material was considered useful to refine the cytological diagnosis.

**Figure 4:**
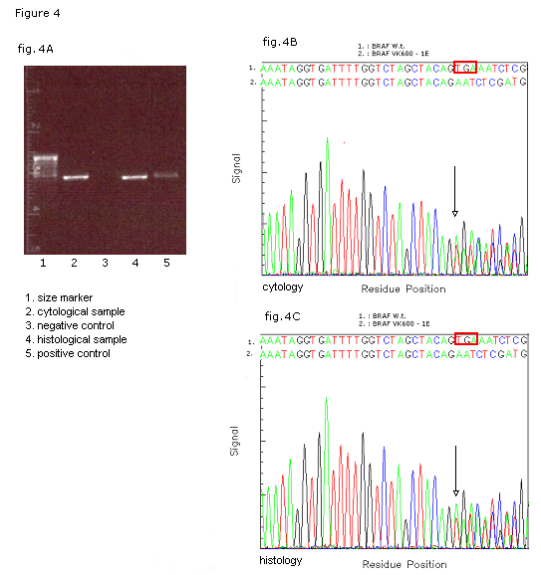
Molecular analysis of BRAF mutational status on matched cyto-histological samples. Electrophoresys of the PCR product of *BRAF *exon (A). Sequencing profile/fluorograph of the matched cytological (B) and histological (C) samples with the respective nucleotide (Nt) sequences of WT (1 BRAF^WT^) and mutated (2 BRAF^VK600-1E^) alleles. The box red in wild-type sequence shows the deleted nucleotide triplet TGA 1799 – 1801.

For the analysis of BRAF mutational status DNA was purified from the thyroid cells suspended in RNAlater by using the QIAAMP DNA Mini Kit following the manufacturer's instructions (QIAGEN Inc). The complete coding sequence of exon 15 of BRAF was amplified by polymerase chain reaction; primers sequence: BRAF exon 15 [forward]: 5'-CTCATCCTAACACATTTCAAGCC-3'; BRAF exon 15 [reverse]: 5'-CTATAGTTGAGACCTTCAATGACTTTC-3'). Appropriate positive (DNA from the neoplastic FRO cell line) and negative controls were included. The PCR product underwent elctrophoresis in agarose gel at the 100 V for 30 minutes (Fig. 4A) and it was analyzed for mutation by direct sequencing with a prior enzymatic purifying treatment and subsequent automatic Sanger sequencing on both strands. A triplet deletion of the coding nucleotides 1799 to 1801 (TGA 1799–1801 deletion) was detected (Fig. 4B). On the basis of the cytological findings and of the molecular results, and in consideration of the reported strong association between the TGA 1799–1801 deletion and the solid variant of PTC, the pre-operative diagnosis was refined and a total thyroidectomy was recommended.

**Figure 5:**
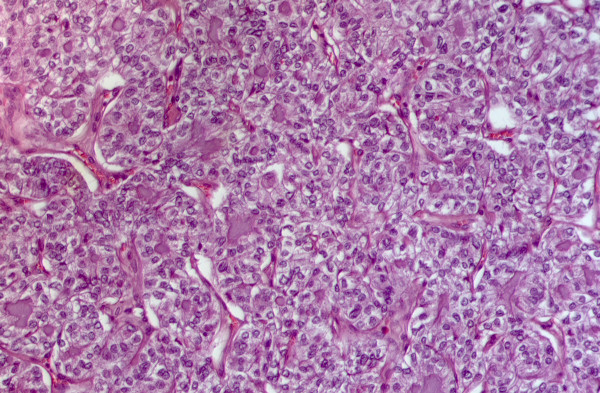
Matched histological sample of papillary carcinoma solid variant. Note the solid architecture of this carcinoma traversed by delicate fibrous septa that delimitates islands and sheets of neoplastic cells (H&E staining 10×)

**Figure 6:**
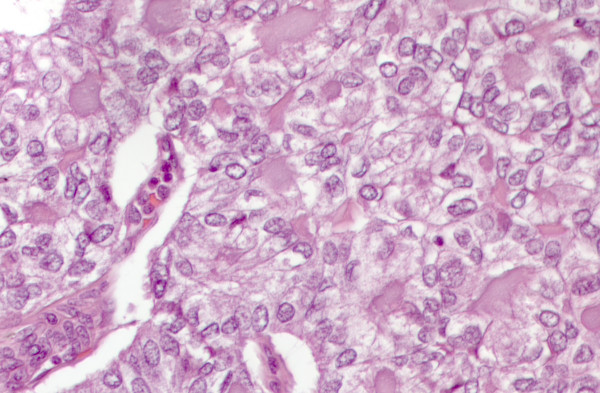
Matched histological sample of papillary carcinoma solid variant. At higher magnification note the nuclear features of the neoplastic cells leading to the morphological diagnosis of PTC in its solid variant. (H&E staining 25×)

Macroscopic examination of the resected specimen showed in the right lobe a 19 mm, well-delimited, non encapsulated lesion, nearly exclusively composed by solid sheets of tumour cells with typical nuclear features of papillary carcinoma (Fig. 5). Nuclear pleomorphism and tumour cell necrosis were not observed; the histological diagnosis of solid variant of papillary thyroid carcinoma was made (Fig. 6). DNA was purified from tumour paraffin embedded block by using the QIAAMP DNA Mini Kit (QIAGEN Inc.) and the same molecular analysis above described for the cytological sample was applied; the TGA 1799–1801 deletion was detected also on the matched histological specimen (Fig. 4C).

## Conclusion

Modern cytology can exploit reliable molecular markers to refine the microscopic diagnosis. In the present study we report a combined cytological and molecular diagnosis of solid variant of papillary thyroid carcinoma. The cytology alone was not conclusive; the little amount of colloid, the elevated cellularity raised the suspect of a neoplasm; however only a slight degree of nuclear changes were observed: grooves and micronucleoli were sporadic, whereas intranuclear inclusions were not observed. The on-site evaluation of the Diff-Quick stained smear was very useful to specify the need of an additional pass to obtain material for *BRAF *molecular analysis. This is an ideal marker in thyroid cytology since it is highly specific for PTC [4]. Furthermore the close relationship between the type of *BRAF *mutation and the histological type of PTC was useful to predict on cytological samples the final precise diagnosis. The *BRAF *triplet deletion TGA 1799–1801 is the hallmark of the solid variant of PTC, having been reported only in this variant [12,13]. Here we first report the detection of this molecular abnormality on FNC samples and we conclude that the *BRAF *triplet detection is of value as a predictive marker of solid variant on subsequently resected PTC.

## Competing interests

The author(s) declare that they have no competing interests.

## Authors' contributions

GT conceived the study and wrote the manuscript; RM and UM carried out the molecular study; IC evaluated the cytological sample; AF evaluated the results; MA evaluated the histological sample; LP participated in the design of the study. All authors read and approved the final manuscript.

## Consent

Written informed consent was obtained from the patient for publication of this case report and any accompanying images. A copy of the written consent is available for review by the Editor-in-Chief of this journal.
